# Clinical Benefits and Risks of Haptoglobin Therapy in Hemolytic Disorders of Diverse Etiologies: A Systematic Review

**DOI:** 10.7759/cureus.100398

**Published:** 2025-12-30

**Authors:** Mohamed Eltayeb Abdelrahman Naiem, Ahmed M.S. Osman, Ahd Atiff Ahmed Abdelghani, Mohammed Elzaki Mohammed Mansoor, Islam Kanada Toto Korea, Ibrahim D Mohammedallayla, Tariq Alkhalifa Yousif Hassan, Mohamed Faisal Elzein Ali

**Affiliations:** 1 General Surgery, Faculty of Medicine, University of Khartoum, Khartoum, SDN; 2 Vascular Surgery, Aberdeen Royal Infirmary - Grampian NHS Trust, Aberdeen, GBR; 3 Intensive Care Medicine, Health Education England, Manchester, GBR; 4 Internal Medicine, University of Gezira, Khartoum, SDN; 5 Internal Medicine, Wexford Hospital, Wexford, IRL; 6 General Practice, University of Medical Sciences and Technology, Khartoum, SDN; 7 Internal Medicine, Sultan Qaboos University Hospital, Muscat, OMN; 8 General Internal Medicine, Midland Regional Hospital, Tullamore, IRL; 9 Police Health Services, Saad Abdullah Academy Hospital, Kuwait City, KWT

**Keywords:** acute kidney injury, burns, cardiac surgery, cell-free hemoglobin, critical care, haptoglobin, hemolysis, systematic review

## Abstract

Hemolytic disorders release cell-free hemoglobin (CFH), a potent nephrotoxin and oxidant. Haptoglobin, an endogenous plasma protein, binds CFH to facilitate its clearance. While haptoglobin therapy is a promising strategy to mitigate CFH-induced organ injury, its clinical efficacy and safety across different hemolytic conditions remain unclear. This systematic review aims to evaluate the clinical benefits and risks of haptoglobin therapy in patients with hemolytic disorders of diverse etiologies.

A systematic literature search was conducted in PubMed, Scopus, Web of Science, and Embase from 2015 to 2025, following PRISMA guidelines. Studies reporting on haptoglobin therapy (administered or endogenous) and clinical outcomes in patients with hemolysis were eligible. The risk of bias was assessed using the ROBINS-I tool. A narrative synthesis was performed due to study heterogeneity. Five observational studies were included, involving cardiac surgery, severe burn, and acute respiratory distress syndrome (ARDS) patients. In cardiac surgery, haptoglobin administration was associated with a significant reduction in postoperative acute kidney injury (AKI) (adjusted OR = 0.54). In contrast, no clinical benefit was observed in severe burn patients. Consistently, low endogenous haptoglobin levels were a strong, independent predictor of AKI and major adverse kidney events (MAKE) across all clinical settings. No significant adverse events were directly attributed to haptoglobin administration. The benefit of haptoglobin therapy is context-dependent, demonstrating significant renal protection in cardiac surgery but not in severe burns. Low endogenous haptoglobin is a robust biomarker for AKI risk. These findings support a targeted, biomarker-guided approach to haptoglobin therapy, rather than universal application. Future randomized trials are needed to confirm efficacy in selected high-risk populations.

## Introduction and background

Hemolytic disorders comprise a heterogeneous group of conditions, characterized by premature red blood cell destruction and the subsequent release of cell-free hemoglobin (CFH) into the circulation [1]. Intravascular hemolysis exposes tissues to unbound hemoglobin, a highly reactive molecule that promotes oxidative stress, nitric oxide scavenging, endothelial dysfunction, and downstream complications, such as vasculopathy and acute kidney injury (AKI) [2,3]. The clinical relevance of these processes is increasingly recognized across diverse hemolytic etiologies, including inherited, immune-mediated, and iatrogenic forms.

Multiple endogenous defense mechanisms exist to limit hemoglobin- and heme-mediated toxicity, including scavenging pathways involving haptoglobin, hemopexin, ferritin, and macrophage-mediated clearance. Among these, haptoglobin binding to free hemoglobin represents the primary, and most efficient, first-line defense in plasma, forming stable complexes that are rapidly removed through CD163-mediated uptake by macrophages [4]. When hemolysis overwhelms this system, or circulating haptoglobin becomes depleted, excess free hemoglobin persists, amplifying vascular and inflammatory injury. This pathophysiological framework has positioned hemoglobin-scavenging strategies as attractive therapeutic targets in translational and clinical research.

Exogenous haptoglobin replacement has therefore emerged as a potential intervention to restore hemoglobin-scavenging capacity, reduce oxidative injury, and mitigate end-organ damage [5]. Experimental studies demonstrate protective effects on renal and vascular function, while early clinical experiences suggest possible benefits in conditions such as sickle cell disease, autoimmune hemolytic anemia, and transfusion-related hemolysis [6]. However, translational reviews and emerging consensus highlight that therapeutic success may depend on appropriate patient selection, baseline haptoglobin depletion, and the interplay with parallel heme-scavenging pathways, underscoring the need for evidence-based evaluation.

Despite growing interest, the clinical efficacy, safety profile, and applicability of haptoglobin therapy across hemolytic disorders remain incompletely defined. Concerns regarding dosing strategies, immunogenicity, adverse events, and interindividual variability persist, and the available data are fragmented across heterogeneous study designs and disease contexts. Accordingly, this systematic review aims to synthesize current clinical evidence on haptoglobin therapy, focusing on therapeutic outcomes, laboratory and clinical endpoints, and reported risks, to clarify its potential role in the management of hemolytic disorders, and to identify critical gaps for future investigation.

## Review

Methodology

Study Design and Protocol

This systematic review was conducted in accordance with the Preferred Reporting Items for Systematic Reviews and Meta-Analyses (PRISMA) guidelines [7], to ensure methodological rigor and transparency. A predefined protocol was developed prior to study initiation, specifying the research question, eligibility criteria, search strategy, outcomes of interest, and data extraction procedures, to minimize bias and enhance reproducibility.

Eligibility Criteria

Study selection was guided by the PICOS framework [8]. The population (P) included patients of any age or sex diagnosed with hemolytic disorders of diverse etiologies, including autoimmune hemolytic anemia, sickle cell disease, and transfusion-associated hemolysis. The intervention (I) of primary interest was exogenous haptoglobin administration (haptoglobin therapy), regardless of formulation, dose, route, or duration. Studies evaluating endogenous haptoglobin levels or genetic variants, without therapeutic administration, were excluded from the primary analysis but were considered during screening to contextualize disease severity and mechanistic rationale.

Comparators (C) included standard care, placebo, or alternative therapeutic approaches. Outcomes (O) comprised clinical benefits (e.g., reduction in hemolysis-related complications), laboratory markers of hemolysis, adverse events, and effects on morbidity or mortality. Eligible study designs (S) included randomized controlled trials, non-randomized interventional studies, and observational studies reporting original clinical data on haptoglobin therapy.

Only peer-reviewed studies published in English between January 1, 2015, and May 31, 2025, were included to ensure contemporary clinical relevance. Gray literature, conference abstracts, editorials, narrative reviews, animal-only studies, and non-English publications were excluded due to limited methodological detail, lack of peer review, and feasibility constraints related to accurate data extraction and interpretation.

Information Sources and Search Strategy

A comprehensive literature search was conducted in PubMed, Scopus, Web of Science, and Embase. The final database searches were performed on May 31, 2025, covering publications from January 1, 2015, to May 31, 2025. Reference lists of included studies and relevant review articles were manually screened to identify additional eligible publications.

Search strategies were adapted appropriately for other databases and are provided in Appendix 1.

Study Selection

All retrieved records were imported into EndNote X9 (Clarivate, Philadelphia, PA, USA), and duplicate citations were removed. Two reviewers independently screened titles and abstracts against the eligibility criteria, followed by full-text assessment of potentially relevant articles. Disagreements were resolved through discussion, with arbitration by a third reviewer when necessary.

Data Collection Process

Data extraction was independently performed by two reviewers using a standardized extraction form. Extracted variables included study characteristics, patient demographics, hemolytic disorder etiology, details of haptoglobin therapy (dose, route, and duration), comparator, follow-up duration, clinical and laboratory outcomes, adverse events, and mortality data. Any discrepancies were resolved through consensus.

Risk of Bias Assessment

The methodological quality of included non-randomized studies was assessed using the ROBINS-I tool [9], which evaluates bias across seven domains, including confounding, participant selection, intervention classification, deviations from intended interventions, missing data, outcome measurement, and selective reporting.

Data Synthesis

Given the substantial heterogeneity in study designs, therapeutic regimens, outcome measures, and follow-up durations, a quantitative meta-analysis was not feasible. Therefore, findings were synthesized narratively, focusing on clinical efficacy, laboratory effects, safety outcomes, and mortality. This approach allowed for structured comparison while accounting for clinical and methodological diversity.

Results

Study Selection Process

**Figure 1:**
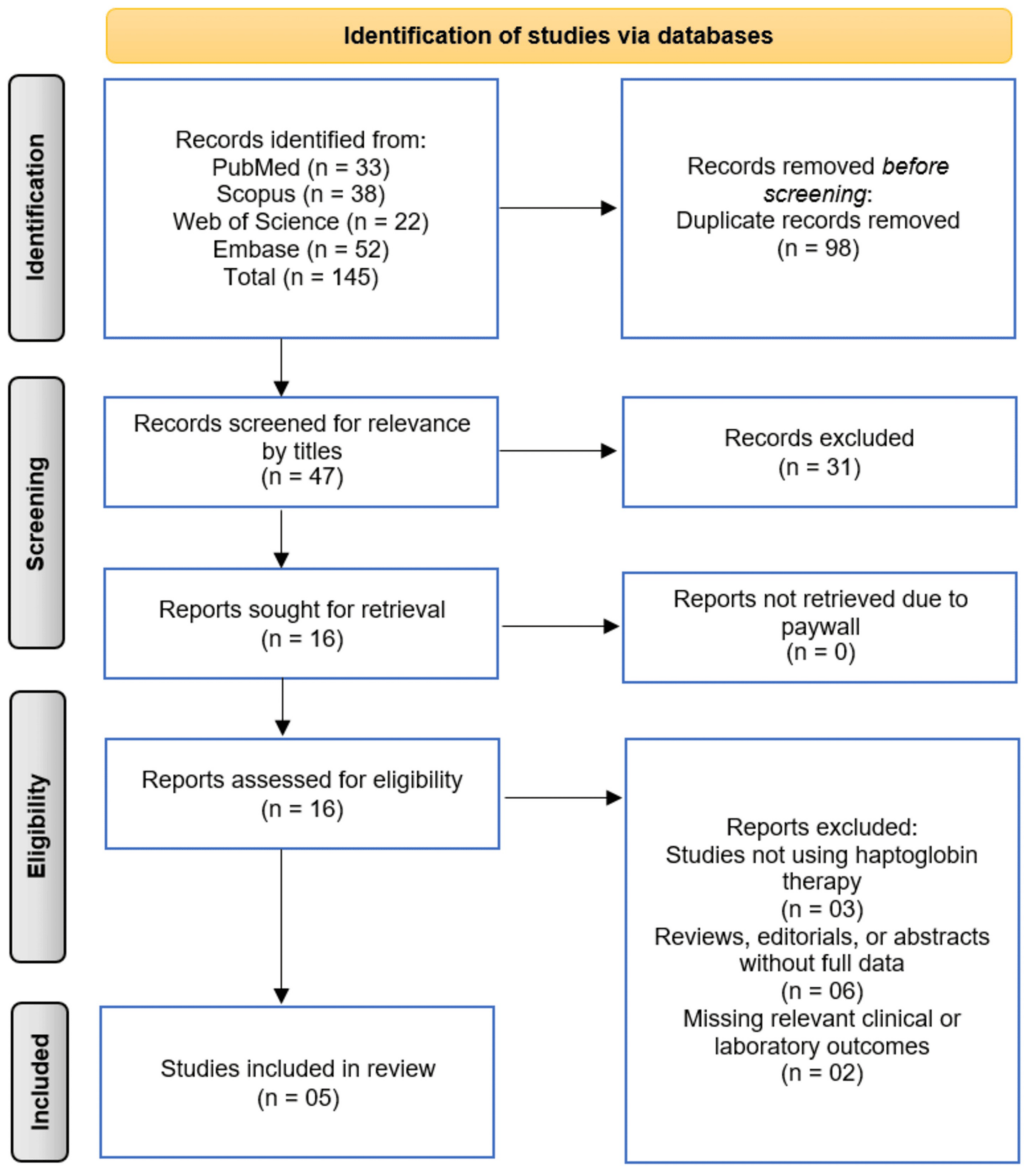
PRISMA Flowchart of Studies Selection Image Credit: Page et al. [7]

The systematic search across four electronic databases (PubMed, Scopus, Web of Science, and Embase) initially identified 145 records. After the removal of 98 duplicate records, 47 unique publications remained for screening. The title screening of these 47 records led to the exclusion of 31 that were not relevant to the review's focus. The full texts of the remaining 16 articles were sought for retrieval, and all were successfully obtained. Upon detailed eligibility assessment, 11 of these articles were excluded for the following reasons: three studies did not involve haptoglobin therapy; six were reviews, editorials, or conference abstracts without full data; and two were excluded due to missing relevant clinical or laboratory outcomes. This rigorous selection process culminated in five studies that met all inclusion criteria and were subsequently included in the qualitative synthesis of this systematic review (Figure 1) [10-14].

Study Characteristics

A total of five studies [10-14] were included in this systematic review, investigating the role of haptoglobin - either through therapeutic administration or endogenous measurement - in patients with hemolytic disorders of diverse etiologies. The characteristics of these studies are summarized in Table 1. The included studies were published between 2017 and 2022 and encompassed a variety of clinical settings, including cardiac surgery [10,14], severe burns [11,12], and acute respiratory distress syndrome (ARDS) requiring extracorporeal membrane oxygenation (ECMO) [13]. Sample sizes ranged from 74 [14] to 3,223 [11]. Three studies employed a retrospective observational design, utilizing propensity score-matched analysis to enhance comparability between groups [10-12]. One study was a prospective observational investigation [14], and another was an analysis of a multicenter ARDS cohort [13]. The primary outcomes consistently focused on kidney injury, including the incidence of AKI, as defined by various criteria (e.g., KDIGO and AKI Network), the requirement for renal replacement therapy (RRT), and major adverse kidney events (MAKE).

**Table 1:** Characteristics of Included Studies AKI, acute kidney injury; MAKE, major adverse kidney events; ARDS, acute respiratory distress syndrome; VV-ECMO, venovenous extracorporeal membrane oxygenation

Author (Year)	Country/Setting	Study Design	Sample Size (n)	Population/Hemolytic Disorder Type	Intervention (Haptoglobin Dose, Route, Duration)	Comparator/Control	Follow-Up Duration	Primary Outcomes Reported
Kubota et al. (2017) [10]	Japan (single-center, cardiac surgery setting)	Retrospective observational study (propensity score-matched analysis)	1,326 patients (260 received haptoglobin; 1,066 did not)	Adult cardiac surgery patients with hemolysis induced by cardiopulmonary bypass	Intraoperative administration of haptoglobin	Patients not receiving haptoglobin	Postoperative period	Incidence of postoperative AKI based on AKI Network criteria; adjusted odds ratio for AKI risk
Tagami et al. (2019) [11]	Japan (nationwide, 618 hospitals)	Retrospective cohort study using propensity score-matched analysis	3,223 total (263 haptoglobin; 2,960 control; 185 pairs after matching)	Patients with severe burns (burn index ≥ 10) are prone to hemolysis-induced AKI	Haptoglobin administered within 2 days of hospital admission	No haptoglobin administration	28 days (in-hospital follow-up)	Requirement for renal replacement therapy, 28-day in-hospital mortality, ventilator-free days, and length of hospital stay
Dépret et al. (2017) [12]	France/Burn Critical Care Unit, Tertiary Center	Retrospective single-center cohort study	130	Critically ill burn patients with intravascular hemolysis	No therapeutic intervention; plasma haptoglobin measured at admission	Comparison between patients with undetectable vs. detectable plasma haptoglobin at admission	Outcomes assessed during ICU stay	MAKE and AKI occurrence; association of undetectable haptoglobin with increased risk of MAKE and AKI
Graw et al. (2022) [13]	Germany (multicenter ARDS cohort)	Observational cohort study	273 (selected from 1044 ARDS patients)	Critically ill patients with ARDS on VV ECMO; hemolysis indicated by circulating cell-free hemoglobin (CFH)	Not administered; study assessed endogenous haptoglobin concentrations (target cutoff >2.4-2.7 g/L)	Patients with low vs. moderate vs. high CFH concentrations	During ECMO initiation and follow-up for AKI development	Incidence of KDIGO stage 3 AKI; association of haptoglobin levels with protection from CFH-associated AKI
Hokka et al. (2021) [14]	Japan/Public teaching hospital	Prospective, observational	74	Adult patients undergoing valvular and aortic surgery requiring cardiopulmonary bypass; no chronic renal failure	None (observational study; haptoglobin measured perioperatively)	None	Perioperative to postoperative day 1	Postoperative AKI and associations with perioperative free hemoglobin and haptoglobin levels

Clinical Benefits of Haptoglobin Therapy and Endogenous Levels

Interventional studies (haptoglobin administration): Evidence from interventional studies evaluating exogenous haptoglobin administration demonstrates variable clinical benefit, depending on patient population and clinical context. In a large retrospective cohort of patients undergoing cardiac surgery, Kubota et al. [10] reported that intraoperative haptoglobin administration was independently associated with a reduced risk of postoperative AKI, defined according to KDIGO criteria during the postoperative period. The adjusted odds ratio for AKI was 0.54 (p = 0.029), with absolute event numbers and confounders included in the multivariable model summarized in Table 2.

**Table 2:** Clinical Benefits and Risks of Haptoglobin Therapy AKI, acute kidney injury; MAKE, major adverse kidney events; ARDS, acute respiratory distress syndrome; VV-ECMO, venovenous extracorporeal membrane oxygenation; CHF, cell-free hemoglobin; CPB, cardiopulmonary bypass

Study (Year)	Clinical Benefits	Laboratory Improvements	Adverse Events/Risks	Mortality/Morbidity Impact	Key Finding
Kubota et al. (2017) [10]	Reduced incidence of postoperative AKI following cardiac surgery	Not directly reported; presumed reduction in nephrotoxic effects of free hemoglobin through fHb scavenging	No significant adverse events reported related to haptoglobin administration	Lower morbidity reflected by decreased AKI occurrence; mortality not reported	Intraoperative haptoglobin administration was independently associated with a significantly reduced risk of AKI after cardiac surgery (adjusted OR = 0.54, p = 0.029)
Tagami et al. (2019) [11]	No significant clinical benefit observed in reducing AKI requiring renal replacement therapy among severe burn patients	No laboratory improvements reported; haptoglobin did not demonstrate measurable biochemical efficacy in this cohort	No major adverse events or safety concerns specifically reported; however, therapy did not confer protective effects	No significant difference in 28-day mortality (46.5% vs 42.7%), ventilator-free days, or hospital stay between haptoglobin-treated and control groups	Haptoglobin therapy did not reduce renal replacement therapy, improve survival, or enhance clinical recovery in patients with severe burns
Dépret et al. (2017) [12]	Potential for kidney protection through correction of hemolysis-related free hemoglobin toxicity	Low or undetectable plasma haptoglobin levels were associated with a higher risk of AKI and MAKE, indicating that adequate haptoglobin levels may reflect better hemolytic control	Not directly assessed; however, undetectable haptoglobin was linked to increased risk of AKI, suggesting possible renal risk when levels are low	ICU mortality was 25%; undetectable haptoglobin independently predicted higher morbidity via increased MAKE and AKI incidence	Undetectable plasma haptoglobin at admission independently predicted AKI and MAKE; findings support the possible benefit of haptoglobin-guided therapy in critically ill burn patients to reduce kidney complications
Graw et al. (2022) [13]	Protective effect of haptoglobin against CFH-associated AKI	Higher plasma haptoglobin concentrations (>2.7 g/L in moderate CFH, >2.4 g/L in high CFH) associated with reduced risk of AKI	AKI risk increased with higher CFH concentrations	Increased CFH concentrations associated with higher AKI incidence (three- to five-fold risk); potential morbidity reduction with adequate haptoglobin levels	Higher haptoglobin levels may protect critically ill ARDS patients on VV ECMO from CFH-associated AKI; identified clinical cutoff values for the protective effect
Hokka et al. (2021) [14]	Not directly evaluated	Haptoglobin levels decrease perioperatively; free hemoglobin levels increase during CPB and normalize by postoperative day 1	Higher free hemoglobin and lower haptoglobin levels are independently associated with increased risk of postoperative AKI	Increased risk of postoperative AKI in 33.8% of patients; no direct mortality data reported	Perioperative low haptoglobin and high free hemoglobin are independent predictors of pAKI after cardiovascular surgery with CPB

Conversely, a nationwide observational study by Tagami et al. [11] in patients with severe burn injury found no significant benefit of haptoglobin therapy. AKI and RRT requirements, defined using standardized clinical criteria during hospitalization, did not differ between the haptoglobin-treated and control groups, nor were differences observed in 28-day in-hospital mortality, ventilator-free days, or length of stay. Absolute event counts and adjusted covariates are provided in Table 2.

Observational biomarker studies (endogenous haptoglobin): Several observational studies examining endogenous haptoglobin levels provide indirect but consistent evidence for a protective role against hemolysis-associated kidney injury. Dépret et al. [12] reported that undetectable plasma haptoglobin at ICU admission was independently associated with an increased risk of AKI and MAKE, with AKI defined using KDIGO criteria during the ICU stay. Absolute event numbers and adjusted confounders are summarized in Table 2.

Similarly, Graw et al. [13] demonstrated that higher circulating haptoglobin concentrations were associated with a lower risk of CFH-associated AKI in critically ill ARDS patients receiving venovenous extracorporeal membrane oxygenation (VV-ECMO). AKI was defined according to KDIGO criteria, and protective haptoglobin cutoff values (>2.7 g/L for moderate CFH and >2.4 g/L for high CFH) were identified. Absolute AKI event counts and adjustment variables are detailed in Table 2.

In line with these findings, Hokka et al. [14] observed that lower perioperative haptoglobin levels were independently associated with an increased risk of postoperative AKI in patients undergoing cardiovascular surgery with cardiopulmonary bypass, using KDIGO-based AKI definitions. Event numbers and multivariable adjustments are summarized in Table 2.

A consolidated summary of clinical outcomes, laboratory effects, absolute event counts, effect estimates, and adjusted confounders across all included studies is presented in Table 2.

Risks and Safety Profile of Haptoglobin Administration

The safety profile of haptoglobin administration was explicitly addressed in several studies. Both Kubota et al. [10] and Tagami et al. [11] reported no significant adverse events or safety concerns specifically attributed to the administration of haptoglobin. However, the study by Tagami et al. [11] highlighted that the therapy did not confer any measurable protective effects in their cohort of burn patients. The risks associated with low endogenous haptoglobin were more prominently featured. The studies by Dépret et al. [12] and Hokka et al. [14] both identified that low or undetectable haptoglobin levels, often coupled with elevated free hemoglobin, were independently associated with a significantly increased risk of renal morbidity, underscoring the risk posed by the body's inability to effectively scavenge CFH.

Summary of Key Findings

The synthesized evidence from the included studies indicates that the potential benefit of haptoglobin is context-dependent. A direct beneficial effect of therapeutic administration was demonstrated in cardiac surgery patients [10], but not in a broader population of severe burn patients [11]. Conversely, multiple observational studies consistently found that low endogenous haptoglobin is a strong predictor of subsequent AKI across diverse critical care and surgical settings [12-14]. This suggests that haptoglobin replacement therapy may be most effective in targeted populations, where hemolysis is a primary driver of organ injury and where endogenous levels are insufficient to cope with the hemolytic load.

Risk of Bias Findings

The assessment of methodological quality using the ROBINS-I tool indicated that the majority of the included studies had a low risk of bias. Four of the five studies - Kubota et al. [10], Tagami et al. [11], Dépret et al. [12], and Hokka et al. [14] - were judged to have a low overall risk of bias, with all assessed domains (confounding, participant selection, classification of interventions, deviations from intended interventions, missing data, measurement of outcomes, and selection of reported results) also rated as low. In contrast, the study by Graw et al. [13] was assessed as having a moderate overall risk of bias. This judgment was due to moderate concerns in the domains of bias due to confounding, bias due to deviations from intended interventions, and bias due to missing data, while the remaining domains were rated as low (Table 3).

**Table 3:** Risk of Bias Assessment Using the ROBINS-I Tool Credit: ROBINS-I Tool [9]

Study (Year)	Bias Due to Confounding	Bias in Selection of Participants	Bias in Classification of Interventions	Bias Due to Deviations From Intended Interventions	Bias Due to Missing Data	Bias in Measurement of Outcomes	Bias in Selection of the Reported Result	Overall Risk of Bias
Kubota et al. (2017) [10]	Low	Low	Low	Low	Low	Low	Low	Low
Tagami et al. (2019) [11]	Low	Low	Low	Low	Low	Low	Low	Low
Dépret et al. (2017) [12]	Low	Low	Low	Low	Low	Low	Low	Low
Graw et al. (2022) [13]	Moderate	Low	Low	Moderate	Moderate	Low	Low	Moderate
Hokka et al. (2021) [14]	Low	Low	Low	Low	Low	Low	Low	Low

Discussion

This systematic review synthesized the clinical evidence on exogenous haptoglobin administration and endogenous haptoglobin levels across diverse hemolytic disorders. Overall, the findings highlight a context-dependent association between haptoglobin availability and clinical outcomes, rather than definitive evidence of therapeutic efficacy. Except for one interventional study, most included data are observational, so reported associations should not be interpreted as proof of causation. The results suggest that haptoglobin may be clinically relevant when hemolysis is a dominant and acute driver of organ injury, but its role is likely limited in more complex, multifactorial disease states.

The strongest interventional signal was observed in the cardiac surgery setting, where Kubota et al. [10] reported that intraoperative haptoglobin administration was independently associated with a lower incidence of postoperative AKI. Importantly, this reflects an association within a controlled perioperative context, rather than definitive proof of efficacy. The biological plausibility is nevertheless compelling, as cardiopulmonary bypass induces rapid and substantial release of CFH that can overwhelm endogenous scavenging. Timely haptoglobin supplementation may, therefore, mitigate hemoglobin-mediated oxidative and renal injury, though causal inference remains limited by the study design [15].

In contrast, Tagami et al. [11] observed no association between haptoglobin administration and improved renal or mortality outcomes in patients with severe burns. This divergence emphasizes that hemolysis is only one component of organ injury in such patients, which also involves systemic inflammation, capillary leak, and infection. Under these circumstances, hemoglobin scavenging alone may be insufficient to alter outcomes, especially when hemolysis is ongoing rather than acute. These findings indicate that haptoglobin therapy may be most effective in discrete, high-intensity hemolytic insults, rather than in multifactorial critical illness.

Observational studies examining endogenous haptoglobin levels consistently demonstrated associations between low or depleted haptoglobin and adverse renal outcomes across diverse populations [12-14]. While these findings do not establish causality, they support the biological concept that adequate haptoglobin is a key protective mechanism. Graw et al. [13] proposed clinically relevant cutoff values, introducing the concept of “functional haptoglobin sufficiency.” Dépret et al. [12] similarly suggested that low plasma haptoglobin identifies patients at higher risk of AKI and MAKE. These observations align with mechanistic studies showing that saturation of the haptoglobin-hemoglobin system leads to CFH overflow and vascular toxicity [16,17]. Hokka et al. [14] further reinforced this association in cardiac surgery patients. Collectively, these studies highlight that endogenous haptoglobin may serve as a biomarker for identifying patients who could potentially benefit from targeted replacement therapy, though direct efficacy remains to be established.

Integrating interventional and observational findings provides a nuanced view: Kubota et al. [10] can be interpreted as correcting transient, procedure-induced haptoglobin insufficiency, whereas Tagami et al. [11] reflects administration in a population where hemolysis was a minor or chronic contributor. Comparisons with other hemolytic conditions, such as complement-mediated intravascular hemolysis in paroxysmal nocturnal hemoglobinuria (PNU), show that simple haptoglobin replacement may be ineffective when hemolysis is continuous, contrasting with successful intervention in acute hemolytic transfusion reactions [5,18]. Genetic evidence, including the Hp2-2 phenotype, associated with lower functional haptoglobin, corroborates these observations and highlights interindividual variability in susceptibility to hemolysis-induced organ injury [19].

Beyond haptoglobin, alternative or adjunct strategies are emerging. These include hemopexin supplementation to scavenge free heme, nitric oxide donors to counteract hemoglobin-mediated NO depletion, and antioxidant therapies to limit oxidative stress. Preclinical studies suggest that combining hemoglobin- and heme-scavenging strategies may provide additive or synergistic protection, particularly in severe or sustained hemolysis [15]. Such approaches underscore that haptoglobin therapy may be most effective as part of an integrated strategy, rather than as a standalone intervention.

Knowledge gaps remain regarding optimal dosing, timing, and patient selection. Existing studies primarily evaluated single intraoperative boluses, leaving unanswered questions regarding repeated dosing, plasma kinetics, durability of haptoglobin-hemoglobin complexes, and the influence of haptoglobin genotype. Safety data are limited to short-term observations [10,11], and potential concerns, such as immunogenicity, iron handling, and reticuloendothelial iron accumulation with repeated or chronic administration, require further investigation [20]. Addressing these gaps is essential to translate biological plausibility into clinically meaningful interventions.

The methodological quality of included studies was generally acceptable, with most demonstrating low risk of bias on ROBINS-I assessment [10-12,14], though one study had moderate risk due to confounding and missing data [13]. Nevertheless, the biological plausibility of findings and their consistency across diverse clinical contexts lend support to the observed associations. Future research should prioritize prospective, biomarker-guided trials with standardized outcome definitions and integrated strategies addressing both hemoglobin and heme toxicity.

Limitations

This systematic review has several limitations. First, the number of included studies was small (n = 5), and all were observational, limiting causal inference - particularly for haptoglobin administration - and leaving results susceptible to residual confounding despite multivariable adjustment. Decisions to administer haptoglobin in studies by Kubota et al. [10] and Tagami et al. [11] may have been influenced by unmeasured clinician judgment or institutional protocols. There was also substantial clinical and methodological heterogeneity across studies, including differences in patient populations, timing and dosing of haptoglobin, outcome definitions, and follow-up periods, which precluded quantitative meta-analysis. Additionally, publication bias cannot be excluded, as studies with null or negative findings may be underrepresented.

Studies evaluating endogenous haptoglobin levels [12-14] are inherently associative and cannot establish causality; low haptoglobin may reflect greater disease severity rather than a direct mediator of AKI. Interpretation is further complicated by inter-center variability in haptoglobin assays, including differences in methodology, units, and normal ranges. Moreover, haptoglobin is an acute-phase reactant, with levels influenced not only by hemolysis but also by inflammation, which may confound its use as a biomarker of hemolytic burden. Finally, the generalizability of the findings is limited, as most interventional data were derived from specific contexts, such as cardiac surgery in Japanese cohorts, and may not be directly applicable to other populations or healthcare settings.

## Conclusions

The role of haptoglobin in managing hemolytic disorders is nuanced and context-specific. The administration of haptoglobin demonstrates a clear benefit in reducing AKI following cardiac surgery, a setting characterized by a profound but discrete hemolytic insult. However, this benefit does not extend to the more heterogeneous and complex pathophysiology of severe burns. Across all settings, low endogenous haptoglobin levels consistently emerge as a powerful predictor of renal injury, underscoring its critical role as a physiological buffer against hemolysis. These findings suggest a paradigm shift from non-selective haptoglobin administration to a more targeted, biomarker-guided approach.

Future research should prioritize the conduct of large, multicenter, randomized controlled trials to definitively establish the efficacy of haptoglobin therapy in high-risk cardiac surgery patients. Furthermore, prospective studies are needed to validate the proposed cutoff values for endogenous haptoglobin and to explore the utility of measuring haptoglobin levels for risk stratification and patient selection in other hemolysis-associated critical illnesses.

## Appendices

Appendix 1

**Table 4:** Search Strategy for Each Database

Database	Search String
PubMed	(“Haptoglobin”[Mesh] OR haptoglobin therapy OR haptoglobin replacement OR exogenous haptoglobin) AND (hemolysis OR hemolytic disorders OR intravascular hemolysis OR free hemoglobin) AND (clinical outcomes OR treatment outcome OR adverse events OR morbidity OR mortality)
Scopus	(TITLE-ABS-KEY(haptoglobin OR “haptoglobin therapy” OR “haptoglobin replacement” OR “exogenous haptoglobin”)) AND (TITLE-ABS-KEY(hemolysis OR “hemolytic disorder*” OR “intravascular hemolysis” OR “free hemoglobin”)) AND (TITLE-ABS-KEY(“clinical outcome*” OR “treatment outcome*” OR “adverse event*” OR morbidity OR mortality))
Web of Science	TS=(haptoglobin OR “haptoglobin therapy” OR “haptoglobin replacement” OR “exogenous haptoglobin”) AND TS=(hemolysis OR “hemolytic disorder*” OR “intravascular hemolysis” OR “free hemoglobin”) AND TS=(“clinical outcome*” OR “treatment outcome*” OR “adverse event*” OR morbidity OR mortality)
Embase	(‘haptoglobin’/exp OR ‘haptoglobin therapy’ OR ‘haptoglobin replacement’ OR ‘exogenous haptoglobin’) AND (‘hemolysis’/exp OR ‘hemolytic disorder*’ OR ‘intravascular hemolysis’ OR ‘free hemoglobin’) AND (‘clinical outcome’/exp OR ‘treatment outcome’ OR ‘adverse event’/exp OR morbidity OR mortality)
